# Supplementary material for: Associations between 10-second standing balance tests and mild cognitive impairment in older adults in Colombia: A cross-sectional national survey
Source: PLOS Glob Public Health. 2025 Sep 17;5(9):e0004543. doi: 10.1371/journal.pgph.0004543 (PMC12443304; doi:10.1371/journal.pgph.0004543)
Supplement: Attachment — Submitted filename: Manuscript Number_PGPH-D-25-00809 Review Report.pdf [file pgph.0004543.s002.pdf]

Manuscript Number: PGPH-D-25-00809 Review Report

Title: Associations between 10-second balance tests and mild cognitive impairment in older adults in Colombia: a national survey

Title

Strength

- It clearly indicated; if there were an association between physical balance and cognitive health in older adults
- Study area and study participants clearly indicated; older adults in **Colombia**.
- Type of the study is clearly indicated; **a national survey that is cross sectional design**

Weakness

- 10-second balance tests are not clear; what kind of test is conducted? Specify the exact test or describe it more clearly
- A national survey" is vague—better if it is described as, Cross-sectional analysis or Findings from a national health survey.
- Study design is not clearly indicated "A cross-sectional national survey.

Overall Recommendation: suggested title

Association between single-leg 10-second balance test performance and mild cognitive impairment among older adults in Colombia: A cross-sectional national survey

Abstract

Strength

- It is well-structured; Objectives, Methods, Results, Conclusions clearly written
- It addresses the under researched area particularly in Latin America including Columbia. The **link between physical and cognitive function.**
- The objective is clearly stated and addresses a gap in knowledge *(lack of data in Latin America)*
- Study participants; clearly indicated that is older
- **The result is reported clearly reported;** confidence intervals are appropriate
- The conclusion is concise and highlights the practical implications.

Weakness

- What is the importance of studying this? Why balance test is undertaken? better if one sentence introduction is incorporated

- What was time of the survey? study period is not indicated
- Is the standard aligning with you counties? Can you elaborate or indicate any national guideline or reference in context of Columbia to classify Mild cognitive impairment?
- How the study reported? Cut off point for reporting the finding not indicated under method section (95% CI; P Value)
- How was the test conducted? Separately or at an interval? One person undertakes the three test simultaneously or only one test?
- The result reported only the prevalence of one test; that is feet-together test. What was the overall prevalence of the total sample? Better to include the outcome of the three tests from the total.
- What was the meaning of success in terms of balance test? completing the test without support for full 10 seconds
- Under conclusion which test is more recommended for further study? What is the prevention or intervention needed to be undertaken?

Overall Recommendation: Abstract

- Minor revisions suggested.

Introduction

Strength

- The introduction is well structured; it indicted global and nation burden of dementia. It give emphasize on the importance of simple predictive tools and finally identifies a gap in research specific to Latin America.

Weakness/Area of improvement

Why it is common in Latin America Specially Columbia? It is disproportionally affected than others; are there additional factors that influence it? Support it by additional literatures.

- What prior evidence suggests a link between physical performance, balance, and cognition, to strengthen rationale? Why this specific test could enhance the justification.

Materials and Methods

It provides sufficient information on the sampling, data collection, outcome variable, and ethical procedures

Sampling design is properly described; **multistage probability sampling**

Ethical approval was obtained from appropriate body and shows compliance with ethical standards.

Weakness/Areas of improvement

- The data were old; why it is not published so far? Even if it is recent national survey and may have relevance at this time.
- It does not show the total number of participants included in the analysis in this section.

Overall Recommendation: Materials and Methods

- Minor refinements for flow and completeness; especially inclusion of sample size and clarity around classification of MCI

Dependent variable and Independent variable

- **dependent variable** is well-explained
- The **MMSE** tool is validated by Chilean study; which increase credibility of the study
- The **independent variable** is described step by step, including instructions given to participants.
- What lower score $\leq$12 or High score 19 indicate? Poor or better cognitive performance? better if clearly explained
- How the number of test reported? Passed/fail; how they were classified passed 0, 1, 2, or all 3 balance tests?
-

Statistical Analyses

- It is well-structured and methodologically sounds. It demonstrates an appropriate analytical approach and thoughtful consideration of confounders.

Results

- It is clearly written and effectively summarizes the findings.
- Why **fewer participants** attempted the semi-tandem and tandem tests. What is the reason behind it? for example only 19,527 participants attempted the tandem test
- **Odds ratios with 95% confidence intervals** properly reported

Area of improvement

- It should be clear the reason why **fewer participants** attempted the semi-tandem and tandem tests.
- clarifications of test attempts vs. successful completion should be given to make the finding coherent

Discussion

- It is written in comprehensive manner, well-structured and relevance for policy.
- It contextualizes the findings within existing literature, addresses implications, and thoughtfully discusses strengths and limitations.
