# Supplementary material for: Associations between 10-second standing balance tests and mild cognitive impairment in older adults in Colombia: A cross-sectional national survey
Source: PLOS Glob Public Health. 2025 Sep 17;5(9):e0004543. doi: 10.1371/journal.pgph.0004543 (PMC12443304; doi:10.1371/journal.pgph.0004543)
Supplement: Attachment — Submitted filename: Reviewers Feedback_Manuscript PGPH-D-25-00809.docx [file pgph.0004543.s001.docx]

**Feedback on Manuscript PGPH-D-25-00809**

The manuscript has several notable strengths and holds strong potential for publication, provided the issues under overall comments and recommendations are addressed.

**Some of the strengths:**

### It addresses a valuable area of research in Latin America by exploring the relationship between balance and cognitive impairment in older adults, offering informative results that hold potential application to the prevention of dementia in Colombia and beyond.

### The large sample of 23,443 participants is a plus for the study as it provides robust statistical power. Moreover, the application of a well-adjusted logistic regression model contributes to the strength of the finding by controlling for confounders.

### The methodology is presented, utilising three validated tests: feet-together, semi-tandem, and tandem balance tests that are recognised predictors of cognitive decline, making the study robust and relevant.

**Overall comments and issues recommended to be addressed are as follows:**

### 1. ****Title and Abstract****

### ****The title:** I**t is informative, clear, concise and reflects the study contents

**Abstract:**

- Well-structured and informative.
- It has summarised the study adequately; however, it could be structured better to enhance clarity.
- **On the Problem Investigated, the abstract briefly stated that little is known about the associations between balance and cognition in Latin America. While this is context information, it could be explicit in defining a specific knowledge(research) gap being addressed, and how this study addresses that gap using such a large national representative dataset (23,433) than leaving the reader to infer that the large sample size makes the findings robust and novel.**
- **On the Results, consider stating statistical significance, such as P-value for odds ratios and confidence interval, clearly to strengthen the quantitative summary in the abstract.**

**Example, ‘…Adjusted odds ratios (95% CI) for mild cognitive impairment were 0.43 (0.39–0.48) for the feet-together test, 0.53 (0.46–0.60) for the semi-tandem test, and 0.63 (0.57–0.70) for the tandem test, rather than 0.43 (0.39, 0.48)…**

- **On Conclusion, it has summarised the findings effectively but did not mention the broader public health implications.**

### ****Suggestion: ‘**These findings highlight the potential of balance tests as cost-effective screening tools for cognitive impairment, thereby supporting their integration into Public health initiatives’.**

- **And the abstract** partially addresses the research gap, does not explicitly frame and explain how the study design addresses the gap.
- **Suggestion:** Strengthen the opening sentences to clearly define the knowledge gap and directly connect it to the study’s contribution.

### 2. ****Introduction****

### **The introduction has provided context well,**

### **To be more engaging, I suggest the Author(s) to emphasise the urgency for early detection or specific challenges unique to Latin America(LA), even if just mentioning… ‘*there is a growing need to identify simple cost-effective screening tools in the context of LA...’***

### **As stated under the Abstract, transition the introduction effectively from global statistics to the LA context brings out the gap.**

### ****Suggestion:** ‘Despite the growing burden of dementia in LA, few studies have examined whether the simple balance tests can serve as an early indicator of cognitive impairment in older adults’.**

### The introduction reiterates the study’s objective without refinement.

### ****Suggestion:**** ‘Despite established links between balance and cognition in North America and Europe, research in Latin America remains scarce. This study leverages data from SABE Colombia, a nationally representative survey of older adults, to investigate associations between 10-second balance tests and mild cognitive impairment.’

### **Focus of the study has been clearly articulated, however, there is nowhere in the introduction the aim of the study is directly stated, but rather the links seems to appear in the discussion section. *‘The present study may have important implications for policy and practice in Colombia. Early detection of cognitive impairment can reduce the burden of dementia by permitting timely action from health care providers, family members, and patients’.***

### ****Suggestion:** The Authors to briefly mention( a bridging sentence ) the aim of the study in the context of dementia prevention strategies in Colombia.**

### Before moving to methods, add a final sentence summarising the potential contribution of the study. This will improve the flow.

### 3. ****Methodology****

### **Well described, but specify study design as stated above under the abstract.**

### **Methodology could also benefit from clearly stated ethical considerations and sampling justification.**

### ****Suggestion:** Instead of ‘*Institutional review boards of Universidad de Caldas and Universidad del Valle approved the study*’**. Refine to read** *‘Ethical approval was obtained from Universidad de Caldas and Universidad del Valle approval number XXX (indicate approval ID)’.* This will ensure compliance with international standards.**

### **If there is any assessed, briefly state assumptions checked for logistic regression such as multi-collinearity, goodness of fit, etc.**

### 4. ****Results****

### **Results are logically structured and clearly presented**

### **As indicated above under the abstract, ensure P-values are consistently reported a long side confidence interval in table 2 and in the text.**

### **Also adjust confidence interval in bracket after the odd ratio as indicated under abstract section above.**

### 5. ****Discussion****

### **The findings were well interpreted, however, they can be expanded to include how it informs dementia prevention strategies to strengthen policy implications.**

### ****For example,** the findings can be refined as follows: ‘*These findings highlight the potential value of balance tests as a cost-effective screening tool for detecting cognitive impairment, supporting their inclusion in public health initiatives’***

### **Also recommend to add a brief reflection on the potential mechanism linking balance and cognition.**

### 6. ****References****

### **Relevant references have been cited.**

### **Minor inconsistencies in spacing and line break.**

### 7. Technical Writing: Refinements in Technical Writing

### For clarity, there is a need to simplify the wording in the methodology and results.

### For example, *a multistage probability sampling method ensured participant representation across municipalities, blocks and households’*… instead of *‘Participants were selected through a multistage probability sampling design involving municipalities, blocks, housing units, and households’.*

### 8. Data transparency and ethical compliance:

### While data availability is mentioned, specific access methods are limited. Thus, the authors ought to have explicitly outlined how others can request access. For example, it could have been stated ‘Data requests should be directed to the Colombian Ministry of Health ([repositorio@minsalud.gov.co](mailto:repositorio@minsalud.gov.co)), citing the dataset Salud, Bienestar and Envejecimiento( SABE Colombia)’.

### Mentioning explicitly adherence to STROBE checklist will strengthen the reporting credibility of a cross-sectional study.

### 9. ****Structure and Language****

### **The manuscript is well organised, however, there is a need to improve conciseness and reduce redundancy in the introduction, methodology and results sections.**

### Minor easily fixable inconsistencies in some line spacing and reference wrapping noted.

### **Example of redundancy within the manuscript and suggested refinements:**

### a) ****Abstract and Introduction.****

### **Current text: ‘**Very little is known about the associations between physical performance and cognition in Latin America. The National Survey of Health, Wellbeing and Ageing in Colombia (SABE Colombia, according to its initials in Spanish) is a nationwide study of community-dwelling adults aged 60 years or older. The objective of the present analysis was to investigate associations between 10-second balance tests and mild cognitive impairment in participants in SABE Colombia.’

### Redundancy: The phrase ‘Very little is known about the association between physical performance and cognition in Latin America" repeats the idea stated earlier in the manuscript under the following areas:

### Abstract: Open statement on research gap, on page 2 and information page at the beginning. ‘*Very little is known about the associations between balance and cognition in Latin* America.’

### Introduction: In line 10 on page 3…*’ However, very little is known about the association…in Latin America’.* This statement introduces the gap, but in a broad and somewhat vague manner.

### Conclusion: the phrase is again repeated in opening statement.

### Again, the same idea is stated without adding new information when quoting previous studies linking physical performance and cognition but data on LA older adults being limited.

### b) Methodology:

### ****Sampling strategy****

### **Current text:** ‘Participants were selected using a multistage area probability sampling design and there were four selection stages: municipalities, blocks, housing units, and households. Data were collected across all departments (that is, states) and the final sample was deemed to be representative of the population of older adults living in households in Colombia.’

### ****Redundancy: The mentioning of ‘****four selection stages: municipalities, blocks, housing units, and households.’ is unnecessarily detailed when the next sentence already confirms the sample’s national representativeness.

### ****Suggestion:**** Revise as stated in under technical writing (no.7) above or state as follows: ‘Participants were selected through a four-stage probability sampling process, ensuring representation of older adults across Colombia.’

### c) ****Results (statistical reporting)****

### ****Current text:**** ‘The odds ratio (95% confidence interval) for mild cognitive impairment was 0.43 (0.39, 0.48) for the feet-together test, 0.53 (0.46, 0.60) for the semi-tandem test, and 0.63 (0.57, 0.70) for the tandem test after adjusting for age, sex, education, smoking, civil status, diabetes, and physical activity.’

### ****Redundancy:**** The phrase "after adjusting for age, sex, education, smoking, civil status, diabetes, and physical activity" is repeated multiple times in other sections.

### ****Suggestion:**** ‘Adjusted odds ratios (95% CI) for mild cognitive impairment were 0.43 (0.39–0.48) for the feet-together test, 0.53 (0.46–0.60) for the semi-tandem test, and 0.63 (0.57–0.70) for the tandem test.’ Adjustment details can be reference in methods section.

#### d) ****Discussion (Policy implication)****

### ****Current Text:**** ‘Early detection of cognitive impairment can reduce the burden of dementia by permitting timely action from health care providers, family members, and patients. A recent evaluation of the current state of dementia prevention and care in Colombia made several recommendations for policy and practice in the country, including: the development of a national dementia plan in keeping with World Health Organization guidelines; greater emphasis on education and other social determinants of health; implementation of education campaigns to reduce the stigma associated with cognitive decline; greater access to health care; a multisectoral approach to primary and secondary prevention; and the promotion of research into dementia in the country.’

### ****Redundancy:**** The sentence ‘Early detection of cognitive impairment can reduce the burden of dementia by permitting timely action from health care providers, family members, and patients.’ repeats a well-known concept without contributing new information.

### ****Suggestion:** ‘**Early identification of cognitive impairment is critical for reducing dementia burden. Policy recommendations in Colombia emphasise national dementia planning, social determinants of health, stigma reduction, and expanded access to care, aligning with WHO guidelines.’

#### e) ****General Formatting Enhancements****

### **To ensure clarity and maintain consistency, the use of active voice is recommended to make sentences more direct and engaging throughout the manuscript.**

Below are sections in the manuscript with passive voice and suggested active voices

i. **Abstract**

### Passive1 (under method line 3*): ‘Mild cognitive impairment was defined as a score of 12 or less out of 19…’*

### Suggested Active voice: *The study defined mild cognitive impairment as a score of 12 or less out of 19…*

### Passive2(under method line 4*):* ‘*Balance was assessed using the feet-together test…’*

### Suggested Active voice: The researchers assessed balance using the feet-together test.…

### Passive3 (under method line 5*):* *‘Logistic regression models were adjusted for age, sex, education…’*

### Suggested Active voice: The authors adjusted logistic regression models for age, sex, education…

ii. **Introduction**

### Passive 1 (line 6): ‘…*the cost of dementia is currently estimated to be around US$ 1,158 million…’*

### Suggested Active voice: …Current estimates place the cost of dementia at around US$ 1,158 million…

iii. **Materials and Methods**

**Participants**

### ****Passive 1 (line 3**):** *Participants* ***were selected*** *using a multistage area probability sampling design…’*

### *****Suggested Active voice:***** The researchers selected participants using a multistage area probability sampling design...

### ****Passive 2 (line 6):**** ‘Data **were collected** across all departments…’ *****Suggested Active voice:***** The study team collected data across all departments…

### ****Passive 3( line)** ‘***Trained interviewers conducted face-to-face interviews in participants’* homes…’ *****Suggested Active voice:***** Trained interviewers conducted interviews in participants’ homes…

iv. Dependent variables

### ****Passive (line 8):**** ‘*A score of 12 or less out of 19* ***was used*** *to screen for mild cognitive impairment…’* *****Suggested Active voice:***** The study used a score of 12 or less out of 19 to screen for mild cognitive impairment.

v. **Independent Variable**

### ****Passive(last line):**** ‘A test **was regarded** as successful if…’

### ****Suggested Active voice:**** The team considered a test successful if…

vi. **Covariates**

- **Passive voice** *(repeated multiple times)***:** ‘*Participants* ***were asked*** *about…’ (repeated multiple times).*
- **Suggested Active voice:** The interviewers asked participants about…

vii. **Statistical Analyses**

- **Passive voice(line 1):** ‘*Logistic regression* ***was used*** *to investigate associations…’*
  **Suggested Active voice:** The researchers used logistic regression to investigate associations…
- **Passive voice 2(line 3): ‘**All logistic regression models **were adjusted** for age, sex, education…
- **Suggested active voice:** The authors adjusted all models for age, sex, education…
- **Passive voice 3: ‘***Odds ratios and 95% confidence intervals* ***were calculated****…’*
  **Suggested active voice:** The team calculated odds ratios and 95% confidence intervals…

viii. **Results**

- **Passive voice 1(line 5): *‘****The prevalence of mild cognitive impairment* ***was*** *20% in those who attempted…’*
  **Suggested active voice:** Twenty percent of those who attempted the feet-together test had mild cognitive impairment.

ix. **Discussion**

- **Passive voice 1(line 7): ‘**All cases of mild cognitive impairment **were identified** using the feet-together test...’
  **Suggested active voice: *The*** feet-together test identified all cases of mild cognitive impairment.
- **Passive voice 2( line : ‘***Longitudinal studies* ***are needed*** *to confirm…’*
  **Suggested active voice:** Future research should confirm…

x. **Strengths and limitations**

- **Passive voice 1:** ‘Static balance tests **were used**…”
  **Suggested active voice:** The study used static balance tests…
- **Passive voice 2:** ‘More research **is required**…’
  **Suggested active voice:** Researchers need to conduct more studies

Finally, clarification on causality is recommended as the study, in my view, implies a predictive relationship despite being cross-sectional. Maybe it is good to state something like… ‘*Since our analysis is cross-sectional, causality cannot be inferred. Longitudinal studies tracking balance and cognitive decline are essential to confirm these associations.’*
