# Supplementary material for: Associations between 10-second standing balance tests and mild cognitive impairment in older adults in Colombia: A cross-sectional national survey
Source: PLOS Glob Public Health. 2025 Sep 17;5(9):e0004543. doi: 10.1371/journal.pgph.0004543 (PMC12443304; doi:10.1371/journal.pgph.0004543)
Supplement: Attachment — Submitted filename: Response to Reviewers.docx [file pgph.0004543.s004.docx]

**Associations between 10-second balance tests and mild cognitive impairment in older adults in Colombia: a national survey (PGPH-D-25-00809)**

**Response to Reviewers**

We thank the reviewers for their constructive and insightful comments. The reviewers’ comments and our responses are below. We have made all the changes the reviewers have suggested that relate to the scientific content of our manuscript. We have also made many of the changes that relate to the writing style.

**Reviewer #1**

[Reviewer’s summary] The manuscript has several notable strengths and holds strong potential for publication, provided the issues under overall comments and recommendations are addressed. Some of the strengths: It addresses a valuable area of research in Latin America by exploring the relationship between balance and cognitive impairment in older adults, offering informative results that hold potential application to the prevention of dementia in Colombia and beyond. The large sample of 23,443 participants is a plus for the study as it provides robust statistical power. Moreover, the application of a well-adjusted logistic regression model contributes to the strength of the finding by controlling for confounders. The methodology is presented, utilising three validated tests: feet-together, semi-tandem, and tandem balance tests that are recognised predictors of cognitive decline, making the study robust and relevant.

[Response to reviewer’s summary] We thank the reviewer for their kind words. We also thank the reviewer for recognising the novelty and importance of our work. We welcome the opportunity to improve our manuscript.

[Comment 1] Data availability statement as currently written, does not fully meet PLOS Global Public Health’s open data policy. Thus, compliance statement could have been written as 'The data underlying this study are owned by the Colombian Ministry of Health and cannot be made publicly available due to ethical and legal restrictions protecting participant privacy. Data are available on reasonable request from the Colombian Ministry of Health (repositorio@minsalud.gov.co) for qualified researchers who meet the criteria for access to confidential data.'

[Response 1] The editorial office also asked us to revise the data availability statement, as follows: “The data used in this analysis are available upon reasonable request. Please email the Colombian Ministry of Health to request access to the data: repositorio@minsalud.gov.co. The original name of the study is in Spanish: Salud, Bienestar & Envejecimiento (SABE Colombia). The analysis plan is available from the corresponding author.”

[Comment 2] [Title] It is informative, clear, concise and reflects the study contents.

[Response 2]. Thank you. We endeavour to write succinctly.

[Comment 3] [Abstract] Well-structured and informative. It has summarised the study adequately; however, it could be structured better to enhance clarity. On the Problem Investigated, the abstract briefly stated that little is known about the associations between balance and cognition in Latin America. While this is context information, it could be explicit in defining a specific knowledge (research) gap being addressed, and how this study addresses that gap using such a large national representative dataset (23,433) than leaving the reader to infer that the large sample size makes the findings robust and novel. On the Results, consider stating statistical significance, such as P-value for odds ratios and confidence interval, clearly to strengthen the quantitative summary in the abstract. Example, ‘…Adjusted odds ratios (95% CI) for mild cognitive impairment were 0.43 (0.39–0.48) for the feet-together test, 0.53 (0.46–0.60) for the semi-tandem test, and 0.63 (0.57–0.70) for the tandem test, rather than 0.43 (0.39, 0.48)… On Conclusion, it has summarised the findings effectively but did not mention the broader public health implications. Suggestion: ‘These findings highlight the potential of balance tests as cost-effective screening tools for cognitive impairment, thereby supporting their integration into Public health initiatives’. And the abstract partially addresses the research gap, does not explicitly frame and explain how the study design addresses the gap. Suggestion: Strengthen the opening sentences to clearly define the knowledge gap and directly connect it to the study’s contribution.

[Response 3] In the Objectives section of the revised Abstract, it now says: “Very little is known about the associations between balance and cognition in Latin America. Therefore, the objective of this study was to investigate associations between 10-second standing balance tests and mild cognitive impairment using data from a large nationally representative study of older adults in Colombia.”

In the Results section of the revised manuscript, it now says: “The odds ratio (95% confidence interval) for mild cognitive impairment was 0.43 (0.39, 0.48) for a successful feet-together test, 0.53 (0.46, 0.60) for a successful semi-tandem test, and 0.63 (0.57, 0.70) for a successful tandem test after adjusting for potential confounders [the p-value for each model was highly significant (all p<0.001)].” Please notice that the odds ratio is expressed “after adjusting for potential confounders”.

In the Conclusion section of the revised manuscript, it now says: “There is a need for simple screening tools in Colombia and other countries in Latin America with limited resources. resources. This study suggests that even the simplest of balance tests can be used to identify older adults at risk of mild cognitive impairment in Colombia. Longitudinal studies are needed to confirm these novel and important findings.”

[Comment 4] [Introduction] The introduction has provided context well. To be more engaging, I suggest the Author(s) to emphasise the urgency for early detection or specific challenges unique to Latin America(LA), even if just mentioning… ‘there is a growing need to identify simple cost-effective screening tools in the context of LA...’. As stated under the Abstract, transition the introduction effectively from global statistics to the LA context brings out the gap. Suggestion: ‘Despite the growing burden of dementia in LA, few studies have examined whether the simple balance tests can serve as an early indicator of cognitive impairment in older adults’. The introduction reiterates the study’s objective without refinement. Suggestion: ‘Despite established links between balance and cognition in North America and Europe, research in Latin America remains scarce. This study leverages data from SABE Colombia, a nationally representative survey of older adults, to investigate associations between 10-second balance tests and mild cognitive impairment.’ Focus of the study has been clearly articulated, however, there is nowhere in the introduction the aim of the study is directly stated, but rather the links seems to appear in the discussion section. ‘The present study may have important implications for policy and practice in Colombia. Early detection of cognitive impairment can reduce the burden of dementia by permitting timely action from health care providers, family members, and patients’.

Suggestion: The Authors to briefly mention( a bridging sentence ) the aim of the study in the context of dementia prevention strategies in Colombia. Before moving to methods, add a final sentence summarising the potential contribution of the study. This will improve the flow.

[Response 4] In the Introduction section of the revised manuscript, it now says: “It is important to identify simple screening tools in countries in Latin America with limited resources because many commonly used screening tools are costly and difficult to implement [Patnode et al., JAMA, 2020, 323, 8, 764-785]. It is particularly important to gather evidence from Colombia because the country is so diverse and so difficult to understand after suffering more than 50 years of civil war [Gutiérrez Robledo et al., Age Ageing, 2022, 51, 5; O’Donovan et al., Epidemiology, 2022, 33, 4, e13-e14].” It also says, “The objective of the present analysis was to investigate associations between 10-second standing balance tests and mild cognitive impairment in participants in SABE Colombia. It would have important implications for policy and practice in Colombia if a simple screening tool were found to predict cognitive decline because a five-year delay in onset might halve the prevalence of dementia [Jorm et al., Acta Psychiatr Scand, 1987, 76, 5, 465-79].”

[Comment 5] [Methodology] Well described, but specify study design as stated above under the abstract. Methodology could also benefit from clearly stated ethical considerations and sampling justification. Suggestion: Instead of ‘Institutional review boards of Universidad de Caldas and Universidad del Valle approved the study’. Refine to read ‘Ethical approval was obtained from Universidad de Caldas and Universidad del Valle approval number XXX (indicate approval ID)’. This will ensure compliance with international standards. If there is any assessed, briefly state assumptions checked for logistic regression such as multi-collinearity, goodness of fit, etc.

[Response 5] In the Methods section of the revised manuscript, the cross-sectional study design is now mentioned: “The National Survey of Health, Wellbeing and Ageing in Colombia is a cross-sectional study that is described in detail elsewhere [Gomez et al., Curr Gerontol Geriatr Res, 2016, 2016, 7910205].”

The sampling design is also described in more detail and the reader is directed to the Technical Report that describes the sampling strategy: “Participants were selected using a multistage area probability sampling design and there were four selection stages: municipalities, blocks, housing units, and households [Ortega-Lenis & Mendez, Colomb Med (Cali), 2019, 50, 2, 128-138]. People living in all regions of Colombia were invited to participate, including people living in the four large cities of Bogotá, Medellín, Cali, and Barranquilla [Ortega-Lenis & Mendez, Colomb Med (Cali), 2019, 50, 2, 128-138]. The response rate was around 62 % in urban areas, around 77 % in rural areas, and around 70 % overall [Gomez et al., Curr Gerontol Geriatr Res, 2016, 2016, 7910205].”

The ethical approval is also described with reference to the approval numbers: “Ethical approval was obtained from institutional review boards of *Universidad de Caldas* (CBCS-021-14) and *Universidad del Valle* (09–014 and 011–015) and all participants gave written informed consent.”

We gladly provide more information about the robustness of the models. In the Methods section, it now says: “We used the Wald statistic to test the robustness of each model. We also fitted logit models to check for collinearity among the covariates.” In the Results section, it now says: “The p-value for each model was highly significant (all p<0.001). The Wald statistic for each model was also highly significant (all p<0.001), which suggests that the models are robust and that sound inferences can be made. There was no evidence of collinearity.”

[Comment 6] [Results] Results are logically structured and clearly presented. As indicated above under the abstract, ensure P-values are consistently reported a long side confidence interval in table 2 and in the text. Also adjust confidence interval in bracket after the odd ratio as indicated under abstract section above.

[Response 6] We now report the p-values for the models. In the Results section, it says: “The odds ratio (95% confidence interval) for mild cognitive impairment was 0.43 (0.39, 0.48) for the feet-together test, 0.53 (0.46, 0.60) for the semi-tandem test, and 0.63 (0.57, 0.70) for the tandem test after adjusting for age, sex, education, smoking, civil status, diabetes, and physical activity. The p-value for each model was highly significant (all p<0.001).” In the footnote of Table 2, it now says: “The p-value for each model was also highly significant (all p<0.001).” Please notice that the odds ratio is expressed “after adjusting for potential confounders”.

[Comment 7] [Discussion] The findings were well interpreted, however, they can be expanded to include how it informs dementia prevention strategies to strengthen policy implications. For example, the findings can be refined as follows: ‘These findings highlight the potential value of balance tests as a cost-effective screening tool for detecting cognitive impairment, supporting their inclusion in public health initiatives’. Also recommend to add a brief reflection on the potential mechanism linking balance and cognition.

[Response 7] We describe the implications for policy and practice in greater detail in the revised manuscript: “The present study may have important implications for policy and practice in Colombia and other countries in Latin America with limited resources. Early detection of cognitive impairment can reduce the burden of dementia by permitting timely action from health care providers, family members, and patients [Morley et al., J Am Med Dir Assoc, 2015, 16, 9, 731-9]. A recent evaluation of the current state of dementia prevention and care in Colombia made several recommendations for policy and practice in the country, including: the development of a national dementia plan in keeping with World Health Organization guidelines; greater emphasis on education and other social determinants of health; implementation of education campaigns to reduce the stigma associated with cognitive decline; greater access to health care; a multisectoral approach to primary and secondary prevention; and the promotion of research into dementia in the country [Guerrero Barragán et al., *Análisis situacional del deterioro cognitivo en Colombia.* Available online]. The present study is important because it suggests that cheap and convenient balance tests could be used to identify those older adults in Colombia who would benefit from the policies and practices mentioned in the national report on dementia prevention and care.”

In the original manuscript and the revised manuscript, we identify potential mechanisms linking balance and cognition: “The results of the present study are biologically plausible. For example, vestibular dysfunction would explain why hearing loss may be a risk factor for dementia in middle-aged adults [Livingston et al., Lancet, 2020, 396, 10248, 413-46] and why poor balance may be a risk factor for dementia in older adults [Meunier et al., Age Ageing, 2021, 50, 4, 1342-8; Stephan et al., J Gerontol A Biol Sci Med Sci, 2024, 79, 8]. The ‘hearing system’ of the outer ear and the middle ear and the ‘balance system’ of the inner ear are both connected to the brain via the vestibulocochlear nerve. Good balance depends upon signals and feedback from the vestibular system and the brain and studies have shown that vestibular function declines with age [Agrawal et al., J Vestib Res 2013, 23, 3, 113-7] and that vestibular dysfunction is associated with worse balance and cognitive decline [Smith et al., Curr Opin Neurol, 2024, 37, 1, 83-7].”

[Comment 8] [References] Relevant references have been cited. Minor inconsistencies in spacing and line break.

[Response 8] We have added several references and checked the formatting of the reference list.

[Comment 9] [Technical Writing: Refinements in Technical Writing] For clarity, there is a need to simplify the wording in the methodology and results. For example, a multistage probability sampling method ensured participant representation across municipalities, blocks and households’… instead of ‘Participants were selected through a multistage probability sampling design involving municipalities, blocks, housing units, and households’.

[Response 9] We have reconsidered the Methods and Results sections and have made several minor changes, largely to avoid any suggestion of duplicating our previous manuscripts [O’Donovan et al., Sci Rep, 2020, 10, 1, 17685; O’Donovan et al., Eur Rev Aging Phys Act, 2022, 19, 1, 28]. We have not changed our description of the sampling design because the use of the passive voice is perfectly acceptable in modern English (Butterfield, Fowler’s Concise Dictionary of Modern English Usage. Third Edition. Oxford: Oxford University Press; 2015).

[Comment 10] [Data transparency and ethical compliance] While data availability is mentioned, specific access methods are limited. Thus, the authors ought to have explicitly outlined how others can request access. For example, it could have been stated ‘Data requests should be directed to the Colombian Ministry of Health (repositorio@minsalud.gov.co), citing the dataset Salud, Bienestar and Envejecimiento( SABE Colombia)’. Mentioning explicitly adherence to STROBE checklist will strengthen the reporting credibility of a cross-sectional study.

[Response 10] As suggested, we have changed the data availability statement. In the revised manuscript, it now says: “The data underlying this study are owned by the Colombian Ministry of Health and cannot be made publicly available due to ethical and legal restrictions protecting participant privacy. Data are available on reasonable request from the Colombian Ministry of Health for qualified researchers who meet the criteria for access to confidential data. Requests to access the data should be directed to the Colombian Ministry of Health: repositorio@minsalud.gov.co. The original name of the study is in Spanish: Salud, Bienestar & Envejecimiento (SABE Colombia).”

In the Methods section of the revised manuscript, it now says: “The present analysis was conducted in agreement with the STROBE Statement and contains items that should be included in reports of cross-sectional studies [STROBE, 2024; Available online].”

[Comment 11] [Structure and Language] The manuscript is well organised, however, there is a need to improve conciseness and reduce redundancy in the introduction, methodology and results sections. Minor easily fixable inconsistencies in some line spacing and reference wrapping noted. Example of redundancy within the manuscript and suggested refinements: a) Abstract and Introduction. Current text: ‘Very little is known about the associations between physical performance and cognition in Latin America. The National Survey of Health, Wellbeing and Ageing in Colombia (SABE Colombia, according to its initials in Spanish) is a nationwide study of community-dwelling adults aged 60 years or older. The objective of the present analysis was to investigate associations between 10-second balance tests and mild cognitive impairment in participants in SABE Colombia.’ Redundancy: The phrase ‘Very little is known about the association between physical performance and cognition in Latin America" repeats the idea stated earlier in the manuscript under the following areas: Abstract: Open statement on research gap, on page 2 and information page at the beginning. ‘Very little is known about the associations between balance and cognition in Latin America.’ Introduction: In line 10 on page 3…’ However, very little is known about the association…in Latin America’. This statement introduces the gap, but in a broad and somewhat vague manner. Conclusion: the phrase is again repeated in opening statement. Again, the same idea is stated without adding new information when quoting previous studies linking physical performance and cognition but data on LA older adults being limited.

[Response 11] We feel that a manuscript loses its flow when more than one or two people are involved in drafting it and we humbly ask the reviewer to respect the writing style we have used in the manuscript.

[Comment 12] [Redundancy] Sampling strategy. Current text: ‘Participants were selected using a multistage area probability sampling design and there were four selection stages: municipalities, blocks, housing units, and households. Data were collected across all departments (that is, states) and the final sample was deemed to be representative of the population of older adults living in households in Colombia.’ Redundancy: The mentioning of ‘four selection stages: municipalities, blocks, housing units, and households.’ is unnecessarily detailed when the next sentence already confirms the sample’s national representativeness.

Suggestion: Revise as stated in under technical writing (no.7) above or state as follows: ‘Participants were selected through a four-stage probability sampling process, ensuring representation of older adults across Colombia.’

[And]

Results (statistical reporting) Current text: ‘The odds ratio (95% confidence interval) for mild cognitive impairment was 0.43 (0.39, 0.48) for the feet-together test, 0.53 (0.46, 0.60) for the semi-tandem test, and 0.63 (0.57, 0.70) for the tandem test after adjusting for age, sex, education, smoking, civil status, diabetes, and physical activity.’ Redundancy: The phrase "after adjusting for age, sex, education, smoking, civil status, diabetes, and physical activity" is repeated multiple times in other sections. Suggestion: ‘Adjusted odds ratios (95% CI) for mild cognitive impairment were 0.43 (0.39–0.48) for the feet-together test, 0.53 (0.46–0.60) for the semi-tandem test, and 0.63 (0.57–0.70) for the tandem test.’ Adjustment details can be reference in methods section.

[And]

Discussion (Policy implication) Current Text: ‘Early detection of cognitive impairment can reduce the burden of dementia by permitting timely action from health care providers, family members, and patients. A recent evaluation of the current state of dementia prevention and care in Colombia made several recommendations for policy and practice in the country, including: the development of a national dementia plan in keeping with World Health Organization guidelines; greater emphasis on education and other social determinants of health; implementation of education campaigns to reduce the stigma associated with cognitive decline; greater access to health care; a multisectoral approach to primary and secondary prevention; and the promotion of research into dementia in the country.’ Redundancy: The sentence ‘Early detection of cognitive impairment can reduce the burden of dementia by permitting timely action from health care providers, family members, and patients.’ repeats a well-known concept without contributing new information. Suggestion: ‘Early identification of cognitive impairment is critical for reducing dementia burden. Policy recommendations in Colombia emphasise national dementia planning, social determinants of health, stigma reduction, and expanded access to care, aligning with WHO guidelines.’

[Response 12] Writing style is individualistic and we do not agree that the repetition of words or ideas is inappropriate. Indeed, according to the authoritative *Fowler’s Guide,* the English language is characterised by redundancy and the repetition of words or ideas is acceptable in any discourse (Butterfield, Fowler’s Concise Dictionary of Modern English Usage. Third Edition. Oxford: Oxford University Press; 2015).

[Comment 13] [Passive voice] To ensure clarity and maintain consistency, the use of active voice is recommended to make sentences more direct and engaging throughout the manuscript.

Below are sections in the manuscript with passive voice and suggested active voices. Abstract. Passive1 (under method line 3): ‘Mild cognitive impairment was defined as a score of 12 or less out of 19…’ Suggested Active voice: The study defined mild cognitive impairment as a score of 12 or less out of 19…

[And]

Passive2(under method line 4): ‘Balance was assessed using the feet-together test…’ Suggested Active voice: The researchers assessed balance using the feet-together test.…

[And]

Passive3 (under method line 5): ‘Logistic regression models were adjusted for age, sex, education…’ Suggested Active voice: The authors adjusted logistic regression models for age, sex, education…

[And]

Introduction. Passive 1 (line 6): ‘…the cost of dementia is currently estimated to be around US$ 1,158 million…’ Suggested Active voice: …Current estimates place the cost of dementia at around US$ 1,158 million…

[And]

Materials and Methods. Participants. Passive 1 (line 3): Participants were selected using a multistage area probability sampling design…’ Suggested Active voice: The researchers selected participants using a multistage area probability sampling design...

[And]

Passive 2 (line 6): ‘Data were collected across all departments…’

Suggested Active voice: The study team collected data across all departments…

[And] Passive 3( line) ‘Trained interviewers conducted face-to-face interviews in participants’ homes…’ Suggested Active voice: Trained interviewers conducted interviews in participants’ homes…

[And]

Dependent variables Passive (line 8): ‘A score of 12 or less out of 19 was used to screen for mild cognitive impairment…’ Suggested Active voice: The study used a score of 12 or less out of 19 to screen for mild cognitive impairment.

[And]

Independent Variable. Passive(last line): ‘A test was regarded as successful if…’ Suggested Active voice: The team considered a test successful if…

[And]

Covariates. Passive voice (repeated multiple times): ‘Participants were asked about…’ (repeated multiple times). Suggested Active voice: The interviewers asked participants about…

[And]

Statistical Analyses. Passive voice(line 1): ‘Logistic regression was used to investigate associations…’ Suggested Active voice: The researchers used logistic regression to investigate associations…

[And]

Passive voice 2(line 3): ‘All logistic regression models were adjusted for age, sex, education… Suggested active voice: The authors adjusted all models for age, sex, education…

[And]

Passive voice 3: ‘Odds ratios and 95% confidence intervals were calculated…’

Suggested active voice: The team calculated odds ratios and 95% confidence intervals…

[And]

Results. Passive voice 1(line 5): ‘The prevalence of mild cognitive impairment was 20% in those who attempted…’

Suggested active voice: Twenty percent of those who attempted the feet-together test had mild cognitive impairment.

[And]

Discussion. Passive voice 1(line 7): ‘All cases of mild cognitive impairment were identified using the feet-together test...’ Suggested active voice: The feet-together test identified all cases of mild cognitive impairment.

[And] Passive voice 2( line : ‘Longitudinal studies are needed to confirm…’ Suggested active voice: Future research should confirm…

[And]

Strengths and limitations. Passive voice 1: ‘Static balance tests were used…” Suggested active voice: The study used static balance tests…

[And]

Passive voice 2: ‘More research is required…’ Suggested active voice: Researchers need to conduct more studies

[Response 13] We have reconsidered our work and made three changes. In the Introduction, it now says: “In Colombia alone, it is currently estimated that the cost of dementia is around US$ 1,158 million per year [Mattap et al., BMJ Glob Health, 2022, 7, 4].” In the Methods section, it now says: “The trained interviewers asked participants about the highest level of education they had achieved, and we created three groups: no education; some primary education; and, some secondary education or more.” In the Methods section, it also says: “Finally, the interviewers asked the participants about sport and exercise and about walking.”

We have made all the changes the reviewer has suggested that relate to the scientific content of our manuscript. We have not made all the changes that pertain to the writing style. We reiterate that writing style is individualistic and that, according to the authoritative guide, the use of the passive voice is perfectly acceptable in modern English (Butterfield, Fowler’s Concise Dictionary of Modern English Usage. Third Edition. Oxford: Oxford University Press; 2015).

[Comment 14] Finally, clarification on causality is recommended as the study, in my view, implies a predictive relationship despite being cross-sectional. Maybe it is good to state something like… ‘Since our analysis is cross-sectional, causality cannot be inferred. Longitudinal studies tracking balance and cognitive decline are essential to confirm these associations.’

[Response 14] We agree that temporality is the major consideration when inferring causality. In the revised manuscript, it says: “Temporality is the major concern when inferring causality [VanderWeele et al., Causal Inference and Scientific Reasoning. In: Lash, et al., Modern Epidemiology. 4th ed. Philadelphia: Wolters Kluwer; 2021] and prospective cohort studies with long follow-up times are needed to confirm the associations between balance tests and mild cognitive impairment observed in the present study.” Thanks again for all your comments!

**Reviewer #2**

[Comment 1] [Title] Associations between 10-second balance tests and mild cognitive impairment in older adults in Colombia: a national survey. Title. Strength. It clearly indicated; if there were an association between physical balance and cognitive health in older adults; Study area and study participants clearly indicated; older adults in Colombia. Type of the study is clearly indicated; a national survey that is cross sectional design. Weakness. 10-second balance tests are not clear; what kind of test is conducted? Specify the exact test or describe it more clearly. A national survey” is vague—better if it is described as, Cross-sectional analysis or Findings from a national health survey. Study design is not clearly indicated “A cross-sectional national survey. Overall Recommendation: suggested title Association between single-leg 10-second balance test performance and mild cognitive impairment among older adults in Colombia: A cross-sectional national survey.

[Response 1] We have changed the title to indicate that standing balance tests were used and that this was a cross-sectional study: “Associations between 10-second standing balance tests and mild cognitive impairment in older adults in Colombia: a cross-sectional national survey”

[Comment 2] [Abstract] Strength. It is well-structured; Objectives, Methods, Results, Conclusions clearly written; It addresses the under researched area particularly in Latin America including Columbia. The link between physical and cognitive function. The objective is clearly stated and addresses a gap in knowledge (lack of data in Latin America). Study participants; clearly indicated that is older; The result is reported clearly reported; confidence intervals are appropriate. The conclusion is concise and highlights the practical implications. Weakness; What is the importance of studying this? Why balance test is undertaken? better if one sentence introduction is incorporated. What was time of the survey? study period is not indicated. Is the standard aligning with you counties? Can you elaborate or indicate any national guideline or reference in context of Columbia to classify Mild cognitive impairment? How the study reported? Cut off point for reporting the finding not indicated under method section (95% CI; P Value). How was the test conducted? Separately or at an interval? One person undertakes the three test simultaneously or only one test? The result reported only the prevalence of one test; that is feet-together test. What was the overall prevalence of the total sample? Better to include the outcome of the three tests from the total. What was the meaning of success in terms of balance test? completing the test without support for full 10 seconds. Under conclusion which test is more recommended for further study? What is the prevention or intervention needed to be undertaken? Overall Recommendation: Abstract. Minor revisions suggested.

[Response 2] We have made several minor changes to the Abtract while adhering to the 300-word limit.

The importance of the study relates to the novel location: Colombia in Latin America. The importance of the study also relates to the simple tests used. In the revised manuscript, it now says: “There is a need for simple screening tools in Colombia and other countries in Latin America with limited resources.”

The study period is now indicated in the revised manuscript: “Trained interviewers collected the data in 2015.”

The use of a validated screening tool is now mentioned in the revised manuscript: “Mild cognitive impairment was defined as a score of 12 or less out of 19 on the shorter version of the mini-mental state examination used and validated in the region.”

Confidence intervals and p-values are now indicated in the revised manuscript: “The odds ratio (95% confidence interval) for mild cognitive impairment was 0.43 (0.39, 0.48) for a successful feet-together test, 0.53 (0.46, 0.60) for a successful semi-tandem test, and 0.63 (0.57, 0.70) for a successful tandem test after adjusting for potential confounders [the p-value for each model was highly significant (all p<0.001)].”

We now explain that three increasingly difficult tests were used: “Balance was assessed using three increasingly difficult standing balance tests: the feet-together test, the semi-tandem test, and the tandem test.”

We now state the prevalance of mild cognitive impariment and the number of participants in each test: “The prevalence of mild cognitive impairment was 20% in 23,443 participants who attempted the feet-together test, 16% in 20,943 who attempted the semi-tandem test, and 15% in 19,527 who attempted the tandem test.”

[Comment 3] [Introduction] Strength. The introduction is well structured; it indicted global and nation burden of dementia. It give emphasize on the importance of simple predictive tools and finally identifies a gap in research specific to Latin America. Weakness/Area of improvement. Why it is common in Latin America Specially Columbia? It is disproportionally affected than others; are there additional factors that influence it? Support it by additional literatures. What prior evidence suggests a link between physical performance, balance, and cognition, to strengthen rationale? Why this specific test could enhance the justification.

[Response 3] In the revised manuscript, we explain with reference to the literature that population growth and population ageing explain the predicted increases in dementia in Colombia and Latin America: “The rising burden of non-communicable diseases in Latin America is likely due to population growth and population ageing [Hambleton et al., Lancet Reg Health Am, 2023, 21, 100483]. Indeed, the projected trends in population growth and population ageing are such that the proportion of people living with dementia is expected to increase by around 75% by 2050 in the United Kingdom and elsewhere in western Europe and by more than 250% in Colombia and elsewhere in Latin America [GBD Dementia Forecasting Collaborators, Lancet Public Health, 2022, 7, 2, e105-e25].”

In the revised manuscript, we mention the plausability of the proposed association with reference to the literature: “It is plausible that balance is associated with cognition [Livingston et al., Lancet, 2020, 396, 10248, 413-46; Agrawal et al., J Vestib Res 2013, 23, 3, 113-7; Smith et al., Curr Opin Neurol, 2024, 37, 1, 83-7].” For example, good balance depends on feedback from the vestibular system and studies have shown that vestibular dysfunction is associated with worse balance and cognitive decline [Smith et al., Curr Opin Neurol, 2024, 37, 1, 83-7].” Biological plausability is considered in greater detail in the Discussion.

In the revised manuscript, we also explain that there is a need for simple screening tools in Colombia: “It is important to identify simple screening tools in countries in Latin America with limited resources because many commonly used screening tools are costly and difficult to implement [Patnode et al., JAMA, 2020, 323, 8, 764-785]. It is particularly important to gather evidence from Colombia because the country is so diverse and so difficult to understand after suffering more than 50 years of civil war [Gutiérrez Robledo et al., Age Ageing, 2022, 51, 5; O’Donovan et al., Epidemiology, 2022, 33, 4, e13-e14].”

[Comment 4] [Materials and Methods] It provides sufficient information on the sampling, data collection, outcome variable, and ethical procedures. Sampling design is properly described; multistage probability sampling. Ethical approval was obtained from appropriate body and shows compliance with ethical standards. Weakness/Areas of improvement; The data were old; why it is not published so far? Even if it is recent national survey and may have relevance at this time. It does not show the total number of participants included in the analysis in this section. Overall Recommendation: Materials and Methods. Minor refinements for flow and completeness; especially inclusion of sample size and clarity around classification of MCI Dependent variable and Independent variable; dependent variable is well-explained. The MMSE tool is validated by Chilean study; which increase credibility of the study. The independent variable is described step by step, including instructions given to participants. What lower score <12 or High score 19 indicate? Poor or better cognitive performance? better if clearly explained. How the number of test reported? Passed/fail; how they were classified passed 0, 1, 2, or all 3 balance tests?

[Response 4] The total sample size is now is now mentioned in the Methods section: “Data were collected across all departments (that is, states) and the final sample of 23,694 men and women was deemed to be representative of the population of older adults living in households in Colombia [Gomez et al., Curr Gerontol Geriatr Res, 2016, 2016, 7910205].”

The analytic sample is described in the Results section, which is normal practice: “The present analysis included data from 23,443 of 23,694 (99%) participants in SABE Colombia.”

We now clarify that a score of 12 or less was indicative of mild cognitive impairment: “A score of 12 or less out of 19 was deemed to be indicative of mild cognitive impairment in SABE Colombia [Gomez et al., Curr Gerontol Geriatr Res, 2016, 2016, 7910205].”

We now explain that the participants were encouraged to attempt three increasingly difficult balance tests: “The trained interviewer informed the participant that they would like them to attempt three increasingly difficult balance tests. The interviewer encouraged the participant to attempt each test but emphasised that the participant did not have to attempt a test if they thought it might be unsafe to do so.”

The participant ‘passed’ the test if they maintained the position for 10 seconds: “A test was regarded as successful if the participant maintained the required position for 10 seconds.”

In the Discussion, we recognise that the data were collected in 2015 and may not be representative of the current popuation: “The data were collected in 2015 and may not be representative of the current population of older adults in Colombia” We first started publishing results from the SABE Colombia survey in 2020 (O’Donovan et al., Sci Rep, 2020, 10, 1, 17685).

[Comment 5] [Statistical Analyses] It is well-structured and methodologically sounds. It demonstrates an appropriate analytical approach and thoughtful consideration of confounders.

[Response 5] Thank you!

[Comment 6] [Results] It is clearly written and effectively summarizes the findings. Why fewer participants attempted the semi-tandem and tandem tests. What is the reason behind it? for example only 19,527 participants attempted the tandem test. Odds ratios with 95% confidence intervals properly reported. Area of improvement. It should be clear the reason why fewer participants attempted the semi-tandem and tandem tests. Clarifications of test attempts vs. successful completion should be given to make the finding coherent.

[Response 6] In the Methods section, wenow explain that the participants were encouraged to attempt three increasingly difficult balance tests: “The trained interviewer informed the participant that they would like them to attempt three increasingly difficult balance tests. The interviewer encouraged the participant to attempt each test but emphasised that the participant did not have to attempt a test if they thought it might be unsafe to do so.”

[Comment 7] [Discussion] It is written in comprehensive manner, well-structured and relevance for policy. It contextualizes the findings within existing literature, addresses implications, and thoughtfully discusses strengths and limitations.

[Response 7] Thanks again for considering our work!
