# Supplementary material for: Associations between 10-second standing balance tests and mild cognitive impairment in older adults in Colombia: A cross-sectional national survey
Source: PLOS Glob Public Health. 2025 Sep 17;5(9):e0004543. doi: 10.1371/journal.pgph.0004543 (PMC12443304; doi:10.1371/journal.pgph.0004543)
Supplement: Attachment — Submitted filename: Response_to_Reviewers_auresp_2.docx [file pgph.0004543.s005.docx]

**Associations between 10-second standing balance tests and mild cognitive impairment in older adults in Colombia: a cross-sectional national survey**

**Response to Reviewers**

We thank the reviewers for considering the revised manuscript. We welcome the opportunity to improve our manuscript. The reviewers’ comments and our responses are below.

**Reviewer #1**

[Comment 1] The authors have adequately addressed the previously suggested comments and revisions. Therefore, I consider the manuscript acceptable for publication once the remaining minor redundancies are refined.

[Response 1] Thanks again for considering our work. We gladly address the minor comments described below.

[Comment 2] Introduction. Line/paragraph: 1–5. Issue: Repetitive explanation of population ageing and dementia rise. i.e.— first as a general factor in Latin America (line 2), then again to explain projections (line 3). Suggested action: Combine and streamline for clarity. See this example: 'Cognitive impairment and dementia affect approximately 57 million people globally, with cases expected to exceed 150 million by 2050. In Latin America, projected population growth and ageing are anticipated to increase dementia prevalence by over 250% by 2050, compared to 75% in Western Europe.'

[Response 2] We have revised the Introduction in order to clarify that two different sources of information are used to strengthen the rationale for the study, rather than ‘repetitive sources’ or ‘repetative information’. In the revised manuscript, it now says: “Data from the United Nations suggest that the rising burden of non-communicable diseases in Latin America is due to population growth and population ageing [Hambleton et al., Lancet Reg Health Am, 2023, 21, 100483]. Furthermore, data from the Global Burden of Disease Study suggest that the projected trends in population growth and population ageing are such that the proportion of people living with dementia is expected to increase by around 75% by 2050 in western Europe and by more than 250% in Latin America [GBD Dementia Forecasting Collaborators, Lancet Public Health, 2022, 7, 2, e105-e25].”

[Comment 3] [Introduction] Line/paragraph: 11–13. Issue: Repetition of knowledge gap framing. Suggested action: Merge sentences.

[And] Line/paragraph: 14–18. Issue: Repetitive justification for simple tools in Colombia. Suggested action: Merge rationale

[Response 3] We have revised the Introduction to describe the different study designs that suggest that balance is associated with cognition in the US and the UK. Please notice that we have described different study designs (cross-sectional and longitudinal) and different outcomes (cognitive decline and dementia). Please also be aware that these studies are described in greater detail in the Discussion. In the revised manuscript, it now says: “Cross-sectional studies suggest that simple balance tests can be used to predict cognitive decline in older adults in the United States [Meunier et al., Age Ageing, 2021, 50, 4, 1342-8]. Longitudinal studies also suggest that balance is associated with dementia in older adults in the United States and the United Kingdom [Bullain et al., J Am Geriatr Soc, 2016, 64, 7, 1408-15; Stephan et al., Gerontol A Biol Sci Med Sci, 2024, 79, 8}. However, very little is known about the associations between simple physical performance tests and cognition in Latin America Rendon-Torres et al., Enferm Clin (Engl Ed), 2021, 31, 2, 91-8.].”

[Comment 4] Results. Line/paragraph: 1–6. Issue: Redundant mention of participant numbers. Repeating “23,443 participants” twice within two sentences is redundant. Suggested action: Combine sentence.See this example: 'The analysis included data from 23,443 of 23,694 (99%) SABE Colombia participants. Of these, 23,443 attempted the feet-together test, 20,943 the semi-tandem test, and 19,527 the tandem test, with complete covariate data.'

[Response 4] As suggested, we have revised the relevant sentences: “Table 1 shows participants’ characteristics according to balance test. The present analysis included data from 23,443 of 23,694 (99%) participants in SABE Colombia. All participants attempted the feet-together test, 20,943 participants attempted the semi-tandem test, and 19,527 participants attempted the tandem test and had complete data on the covariates.”

[Comment 5] [Results] Line/paragraph: 7–10. Issue: Repeating prevalence rates already in abstract. Repeating prevalence figures for each test were already stated in the abstract and are repeated verbatim here without adding new context. Suggested Action: Summarize or remove. See this example summarising without repeating: 'As shown in Table 1, prevalence of mild cognitive impairment decreased progressively across the three balance tests.' If you wish to retain figures for clarity, state them once in the text or table, not both.

[Response 5] We have changed the Results section in order to avoid repeating the prevalance rates in the Abstract: “The prevalence of mild cognitive impairment was 20% in those who attempted the feet-together test and was lower in those who attempted the semi-tandem test and those who attempted the tandem test.”

**Reviewer #2**

[Comment 1] The Auther have addressed all the comment provided for him.

Regarding dual submission the author should confirm the manuscript is not under consideration elsewhere.

[Response 1] Thanks again for considering our work. In the Cover Letter to the journal, it states: “The paper and the data have not previously been published, either in whole or in part, and no similar paper is in press or under review elsewhere. We, the authors, have no conflicts of interest. There are no closely related papers.”

[Comment 2] The availability of the data upon request must be clearly indicated. What are the requirements to access the data based on reasonable request from the publisher/Journal? Expected response time. E.g. how many days it requires the author to get/access the data for this analysis. What are the potential barriers to access the data?

[Response 2] We obtained the data from the Colombian Ministry of Health more than five years ago. There have been several changes in administration and decreases in funding in the meantime and we could not possibly speculate how difficult it might be or how long it might take to gain access to the data. We wish we could be more helpful, but Colombia is a relatively bureaucratic and inefficient country. We have amended the Data Availability Statement to indicate that a user agreement is required: “The data used in this analysis are available upon reasonable request, depending on a user agreement. Please email the Colombian Ministry of Health to request access to the data: repositorio@minsalud.gov.co. The original name of the study is in Spanish: Salud, Bienestar & Envejecimiento (SABE Colombia). The analysis plan is available from the corresponding author.”
